# Toll-like Receptor Expression Patterns in the Female Reproductive Tract of Sheep

**DOI:** 10.3390/ani15121704

**Published:** 2025-06-09

**Authors:** Zhibo Wang, Jian Zheng, Hua Yang, Xu Feng, Fengzhe Li, Jing Pang, Xiaolei Yao, Feng Wang, Yanli Zhang

**Affiliations:** 1 Jiangsu Livestock Embryo Engineering Laboratory, College of Animal Science and Technology, Nanjing Agricultural University, Nanjing 210095, China; 2020205010@stu.njau.edu.cn (Z.W.); 2016105032@njau.edu.cn (J.Z.); yanghua004@njau.edu.cn (H.Y.); 2018205026@njau.edu.cn (X.F.); lfz162518@live.com (F.L.); 2016105031@njau.edu.cn (J.P.); yaoxiaolei@njau.edu.cn (X.Y.); caeet@njau.edu.cn (F.W.); 2 Shanghai Animal Disease Prevention and Control Center, Shanghai 201103, China

**Keywords:** Toll-like receptors, Hu sheep, reproductive organs

## Abstract

Toll-like receptors (TLRs) play crucial roles in innate immunity, but their function in reproduction remains poorly understood. This study investigated the expression patterns and localization of TLR1-9 in the reproductive system of Hu sheep and their potential association with prolificacy. All TLRs were expressed in the oviduct, uterus, and ovary, with TLR6 showing significantly higher expression in the oviduct, while TLR3, TLR6, and TLR7 were predominantly expressed in the ovary. Following this initial screening, we focused on TLR2, TLR6, and TLR7 for detailed analysis. Immunohistochemical analysis revealed that TLR2, TLR6, and TLR7 were localized in the luminal epithelium and circular muscle of the oviduct, the luminal and superficial glandular epithelium of the uterus, and in ovarian follicles at all developmental stages. A comparative analysis between high-prolificacy (HP) and low-prolificacy (LP) Hu sheep demonstrated significantly lower TLR2 expression in the reproductive organs of HP sheep, while TLR6 expression was higher and TLR7 expression was lower in HP ovaries compared to LP ovaries. Notably, TLR7 was observed around apoptotic bodies of granulosa cells, suggesting a potential role in follicular development through the regulation of granulosa cell apoptosis. These findings establish a novel link between innate immunity and reproductive function in sheep, suggesting that TLRs, particularly TLR2, TLR6, and TLR7, may serve dual roles as immune sentinels and reproductive regulators influencing ovine fertility.

## Simple Summary

Fertility in farm animals is critical for agricultural productivity, yet we do not fully understand all the biological factors that control it. Our immune system contains special proteins called Toll-like receptors that normally help defend against infections, but they might also play unexpected roles in reproduction. We examined these receptors in the reproductive organs of Hu sheep, a breed known for having multiple lambs per pregnancy. We discovered that these immune receptors are present throughout the female reproductive system, with three specific types showing interesting patterns. Remarkably, sheep that typically produce more lambs had different amounts of these receptors compared to those producing fewer lambs. One receptor type was found near dying cells in the ovaries, suggesting it might help control egg development. This research reveals a surprising connection between the immune system and fertility in sheep. Understanding this relationship could potentially help farmers improve breeding programs and address fertility problems in livestock, ultimately contributing to more sustainable and productive agriculture. Our findings open new possibilities for enhancing reproductive efficiency in sheep through methods that influence immune function.

## 1. Introduction

Toll-like receptors (TLRs) are integral components of the Pattern Recognition Receptor superfamily that detect invading pathogens through recognition of conserved molecular structures known as pathogen-associated molecular patterns (PAMPs). These receptors play a pivotal role in innate immunity, with their specific expression patterns directly linked to the host’s resistance against pathogenic challenges [1,2]. The expression profiles of TLRs have been extensively characterized across diverse species, including goats [3], water buffalos [4], mice [5,6,7], humans [8,9], chickens, and bovines [10,11].

TLRs are encoded by 13 genes in various mammalian species, including goat, sheep, pig, cattle, rabbit, and human [4,12,13,14,15,16]. Notably, distinct expression patterns of TLRs have been observed in the reproductive organs of both mice and rabbits [17,18], suggesting a significant regulatory role in reproductive organ function and fertility. These receptors have been demonstrated to play critical roles in the female reproductive system [19,20]. Ovulation, which encompasses the release of the follicle and the formation of the corpus luteum, involves a coordinated cascade of physiological events that include immune-related signaling [21]. Previous studies have reported that the release of cytokines and immune-modulating factors can promote the survival of the cumulus oocyte complex (COC) and protect it from pathogen-induced damage in the reproductive tract during ovulation by activating TLRs [22,23]. Furthermore, TLR4 has been implicated in modulating immune responses in the uterus and oviduct of female rabbits [24], suggesting its importance in maintaining reproductive tract homeostasis.

Reproductive performance significantly influences the profitability of ovine production, and given that TLRs are expressed in mammalian reproductive systems with crucial roles in reproductive processes [25], understanding their expression patterns in sheep is of considerable importance. Despite this relevance, there is currently a paucity of research examining TLR expression patterns in the reproductive system of Hu sheep, particularly in relation to follicular development and reproductive organ function. Additionally, limited studies have investigated TLR expression and immune responses within the reproductive tract of ewes. We hypothesized that there exists a specific expression pattern of TLRs in the reproductive system of female sheep, and that specific TLRs may be pivotal in influencing fecundity. To test this hypothesis, the present study investigates the expression profiles of TLR gene families in the primary female reproductive organs, including the uterus, oviduct, and ovary of Hu sheep, with a particular focus on individuals exhibiting high versus low prolificacy.

## 2. Materials and Methods

### 2.1. Animal Ethics

All the experiments were carried out under the guidance in animal experiments of Nanjing Agricultural University and approved by the Animal Care and Use Committee of Nanjing Agricultural University, China (approval number: SYXK2022-0031).

### 2.2. Animals, Experimental Design and Sample Collection

Hu sheep ewes were categorized into the high-prolificacy (HP) group (*n* = 3, body weight 53.87 ± 2.98 kg, litter size = 3) and the low-prolificacy (LP) group (*n* = 3, body weight 47.67 ± 1.23 kg, litter size = 1) based on birth records. Synchronous estrus was induced using progesterone vaginal sponges for 10 days followed by 0.2 mg prostaglandin F2α injection.

Upon estrus detection in the second cycle, the ewes were euthanized and the reproductive organs (ovaries, oviducts, uterine horns, cervix, and vagina) were collected. Tissue samples were divided into two portions: one flash-frozen in liquid nitrogen and stored at −80 °C for molecular analyses, and the other fixed in 4% paraformaldehyde in PBS (pH 7.4) for 24 h at 4 °C for histological examination. Ovarian tissues were carefully processed to preserve follicles at different developmental stages. Fixed tissues were dehydrated through an ethanol gradient, embedded in paraffin, and sectioned at 5 μm thickness. All procedures were performed using sterile techniques to prevent cross-contamination.

### 2.3. RNA Extracted and qRT-PCR

Approximately 100 mg of tissue samples was homogenized in 1 mL TRIzol (Invitrogen, Carlsbad, CA, USA) and processed for isolation according to the manufacturer’s instructions. Briefly, total RNA was resuspended in DEPC-treated deionized water, and the RNA concentration and purity were measured by a Nanodrop 2000 spectrophotometric (Thermo Scientific, Waltham, MA, USA). Samples with an OD 260/280 ratio between 1.8 and 2.0 were selected for further analysis. Then, 1 μg of total RNA was reverse-transcribed into complementary DNA (cDNA) using PrimeScript™ RT reagent Kit (Accurate Biotechnology, Changsha, China, Cat No. AG11728) in a 20 μL reaction system under 37 °C for 15 min, followed 85 °C for 5 s for on the T100 Thermal Cycler (186-1096, Bio-Rad, Singapore). The primers for quantitative RT-polymerase chain reaction (qRT-PCR) were designed using Primer 5.0 and listed in Table 1. Glyceraldehyde 3-phosphate dehydrogenase (GAPDH) served as the internal control, and all samples were measured in triplicate. The relative quantification of the target gene (Toll-like Receptor Family) expression levels was conducted using the 2^−ΔΔCt^ method.

### 2.4. Immunohistochemistry

The immunocytochemical analysis was performed using a previously established method [26]. The following primary antibodies were utilized: rabbit anti-TLR7 primary antibody (1:200 dilutions; ab45371, Abcam, Cambridge, MA, USA), rabbit anti-TLR2 primary antibody (1:200 dilutions; D220100, BBI Life Science, Shanghai, China), TLR6 primary antibody (1:200 dilutions; D261448, BBI Life Science, China). A goat anti-rabbit IgG secondary antibody was applied at a dilution of 1:1000 (AP132P, BOSTER, Wuhan, China). The samples were incubated with a streptavidin–biotin complex. The negative controls were treated with Tris-buffered saline (TBS) instead of the primary antibody. Three sections were selected from each organ, and five different areas from each section were analyzed using the Image-Pro Plus 6.0 and microscope (Nikon, Tokyo, Japan) at 400× and 200× magnification.

### 2.5. Statistical Analysis

**Figure 1:**
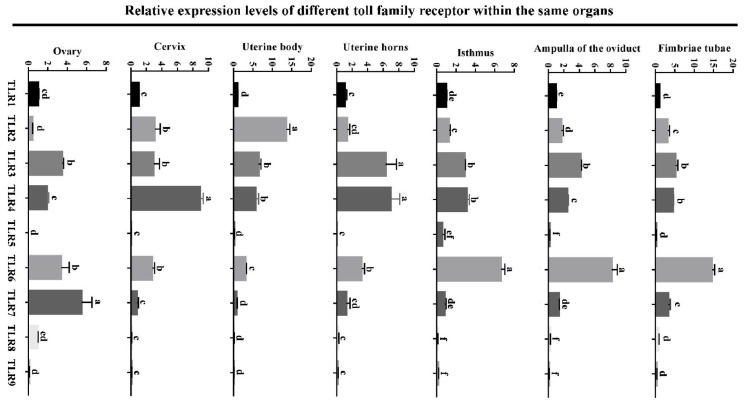
**Different expression of TLRs in various reproductive organs of Hu sheep.** The relative expression levels were normalized to GAPDH, and the results were expressed relative to TLR1 expression group, with different letters representing significant differences (*p* < 0.05).

**Figure 2:**
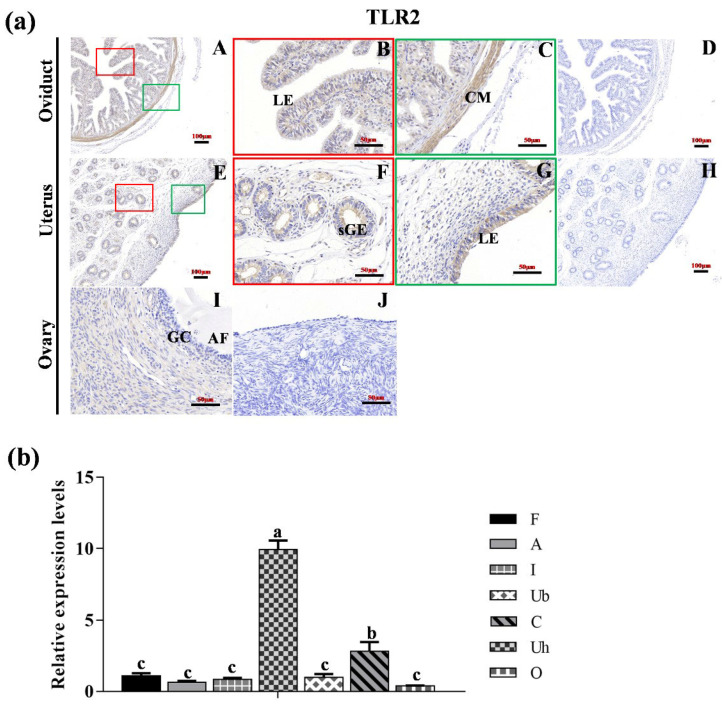
**The location and expression of TLR2 in various reproductive organs of Hu sheep.** (**a**) Immunohistochemical localization of TLR2 proteins in the oviduct (**A**–**D**), uterus (**E**–**H**) and ovary (**I**,**J**) of the Hu sheep. (**D**,**H**,**J**) represent the negative controls for the oviduct, uterus, and ovary, respectively. Magnification: 400× (**B**,**C**,**F**,**G**); 200× (**A**,**D**,**E**,**H**–**J**). LE: luminal epithelium; CM: circle muscle; sGE: superficial glandular; AF: antral follicle; GC: granulosa cell. (**b**) The expression level of TLR2 in different organs of the Hu sheep. The experiment was biologically repeated three times (*n* = 3) and technically repeated three times for each group. The relative mRNA levels were normalized to GAPDH and the results were expressed relative to F group, with different letters representing significant differences (*p* < 0.05). F: fimbria tubae; A: ampulla of the oviduct; I: isthmus; Ub: uterine body; Uh: uterine horn; C: cervix; O: ovary.

**Figure 3:**
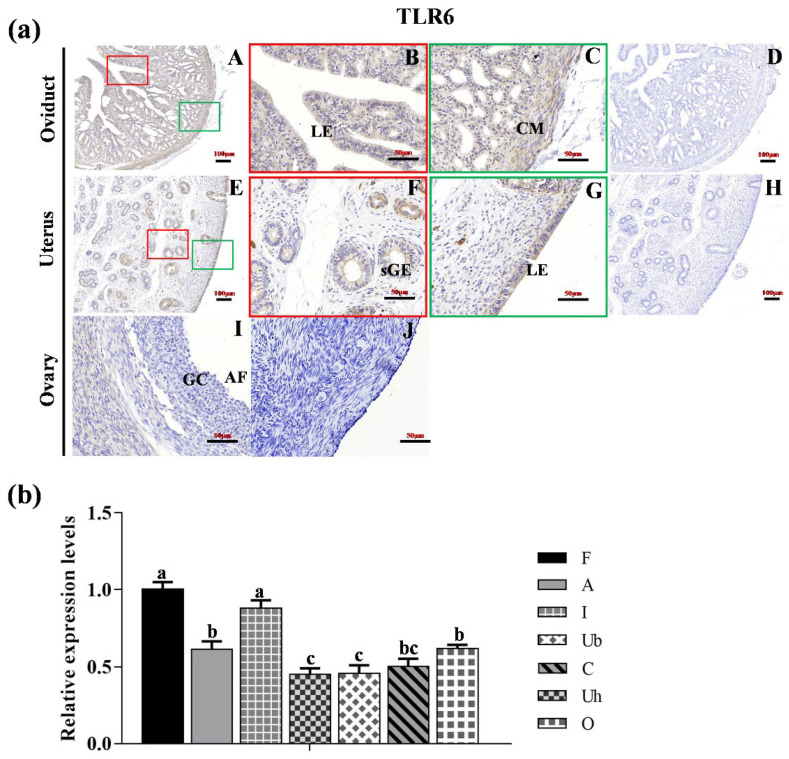
**The location and expression of TLR6 in various reproductive organs of Hu sheep.** (**a**) Immunohistochemical localization of TLR6 proteins in the oviduct (**A**–**D**), uterus (**E**–**H**) and ovary (**I**,**J**) of the Hu sheep. (**D**,**H**,**J**) represent the negative controls for the oviduct, uterus, and ovary, respectively. Magnification: 400× (**B**,**C**,**F**,**G**); 200× (**A**,**D**,**E**,**H**–**J**). LE: luminal epithelium; CM: circle muscle; sGE: superficial glandular; AF: antral follicle; GC: granulosa cell. (**b**) The expression level of TLR6 in different organs of the Hu sheep. The experiment was biologically repeated three times (*n* = 3) and technically repeated three times for each group. The relative expression levels were normalized to GAPDH and the results were expressed relative to C group, with different letters representing significant differences (*p* < 0.05). F: fimbria tubae; A: ampulla of the oviduct; I: isthmus; Ub: uterine body; Uh: uterine horn; C: cervix; O: ovary.

**Figure 4:**
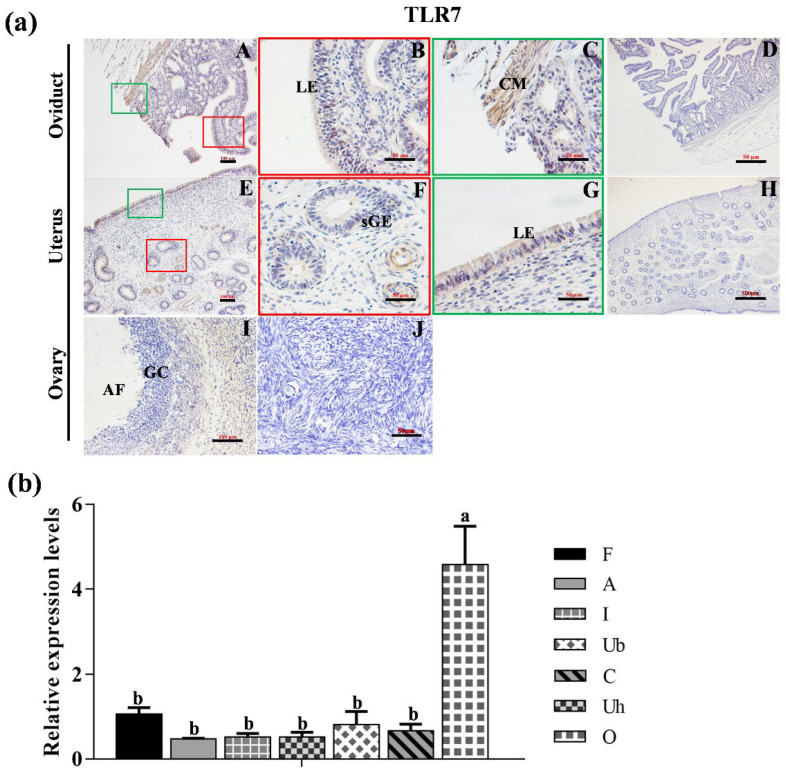
**The location and expression of TLR7 in various reproductive organs of Hu sheep.** (**a**) Immunohistochemical localization of TLR7 proteins in the oviduct (**A**–**D**), uterus (**E**–**H**) and ovary (**I**,**J**) of the Hu sheep. (**D**,**H**,**J**) represent the negative controls for the oviduct, uterus, and ovary, respectively. Magnification: 400× (**B**,**C**,**F**,**G**); 200× (**A**,**D**,**E**,**H**–**J**). LE: luminal epithelium; CM: circle muscle; sGE: superficial glandular; AF: antral follicle; GC: granulosa cell. (**b**) The expression level of TLR7 in different organs of the Hu sheep. The experiment was biologically repeated three times (*n* = 3) and technically repeated three times for each group. The relative expression levels were normalized to GAPDH and the results were expressed relative to C group, with different letters representing significant differences (*p* < 0.05). F: fimbria tubae; A: ampulla of the oviduct; I: isthmus; Ub: uterine body; Uh: uterine horn; C: cervix; O: ovary.

Data were analyzed using one-way ANOVA with the Statistical Package for the Social Sciences (SPSS) 23.0 [18]. For the data presented in Figure 1, Duncan’s multiple comparison was employed to test the differences in TLRs expression across reproductive organs. For the data shown in Figure 2, Figure 3 and Figure 4, Duncan’s multiple comparison test was utilized to evaluate differences in TLR2, TLR6, and TLR7 among various reproductive organs. The *t*-test was used to test the differences between the LP and HP group [27]. Each replicate served as an experiment unit. Data are shown as means ± standard errors (SE), and differences were considered statistically significant at *p* < 0.05.

## 3. Results

### 3.1. Expression Levels of Toll-like Receptor Family in Different Reproductive Organs

As shown in Figure 1, all TLRs (TLR1, 2, 3, 4, 5, 6, 7, 8 and 9) were expressed in the oviduct, uterine and ovary. Notably, TLR6 exhibited significantly higher mRNA levels (*p* < 0.05) in the oviduct compared to the other TLRs. Consistent expression patterns of TLR6 were observed in the fimbriae tubae, ampulla, and isthmus of the oviduct. In the uterus, TLR2, TLR3, and TLR4 displayed distinct expression profiles across the uterine horn, body, and cervix. Overall, TLR5, TLR8, and TLR9 exhibited the lowest expression levels (*p* < 0.05) among all TLRs in the uterus, oviduct, and ovary. In the ovary, the TLR3, TLR6 and TLR7 levels were significantly higher (*p* < 0.05) than those of TLR1, TLR2, TLR4, TLR5, TLR8, and TLR9.

### 3.2. Localization of TLR2, TLR6 and TLR7 in Various Reproductive Organs and mRNA Expression in Hu Sheep Tissues

As depicted in Figure 2a, TLR2 was localized in the luminal epithelium (LE) and circle muscle (CM) of the oviduct, with notably stronger staining observed in the CM compared to the LE (Figure 2a(A–C)). In the uterus, TLR2 was localized in both LE and superficial glandular (sGE) (Figure 2a(E–G)). In the ovary, TLR2 was expressed in ovarian follicle (I). No specific staining was detected in the negative control sections (Figure 2a(D,H,J)). Figure 2b showed the expression level of *TLR2* across different reproductive organs, with the fimbriae tubae serving as a reference. The expression level of *TLR2* in the uterine horn was significantly higher than in the other reproductive organs (*p* < 0.05).

As illustrated in Figure 3a, immunohistochemical analysis revealed that TLR6 protein was predominantly localized in the LE and CM of the oviduct (Figure 3a(A–C)), as well as in the LE and sGE of the uterus (Figure 3a(E–G)). In the ovary, TLR6 exhibited weak expression in the ovarian follicle (Figure 3a(I)). No specific staining was observed in the negative control sections (Figure 3a(D,H,J)). Furthermore, the expression level of *TLR6* in the fimbriae tubae and isthmus were significantly higher compared to those in the other reproductive organs (Figure 3b, (*p* < 0.05)).

As depicted in Figure 4a, TLR7 protein was expressed in the LE and CM of the oviduct, with stronger staining in CM than that in LE (Figure 4a(A–C)). Moreover, TLR7 was localized in both the LE and sGE of the uterus (Figure 4a(E–G)), as well as in the ovarian follicles (I). No specific staining was detected in the negative control sections (Figure 4a(D,H,J). In addition, the mRNA level of *TLR7* in the ovary was significantly higher than in the other reproductive organs (Figure 4b, *p* < 0.05).

### 3.3. Localization of TLR2, TLR6 and TLR7 in Follicles at Different Developmental Stages in Hu Sheep

**Figure 5:**
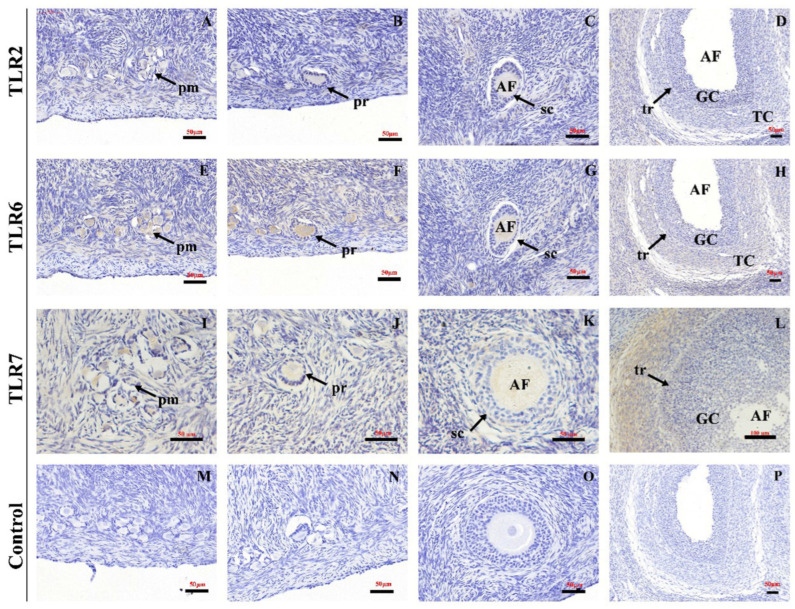
**The location of TLR2, TLR6 and TLR7 in different developmental stages of ovarian follicles of Hu sheep.** Magnification: 400× (**A**–**C**,**E**–**G**,**I**–**K**,**M**–**O**); 200× (**D**,**H**,**L**,**P**). pm: primordial follicle; pr: primary follicle; sc: secondary follicle; tr: tertiary follicle; GC: granulosa cell; TC: theca cell; AF: antral follicle.

In the follicles, TLR2, 6 and 7 were localized in all follicle stages, including the primordial follicle (pm) stage (Figure 5A,E,I), primary follicles (pr) (Figure 5B,F,J), secondary follicles (sc) (Figure 5C,H,M), and tertiary follicles (tr) (Figure 5D,H,L). No specific staining was detected in the negative control sections (Figure 5M–P).

### 3.4. Protein and mRNA Levels of TLR2, TLR6 and TLR7 in Different Tissues of HP and LP Group

**Figure 6:**
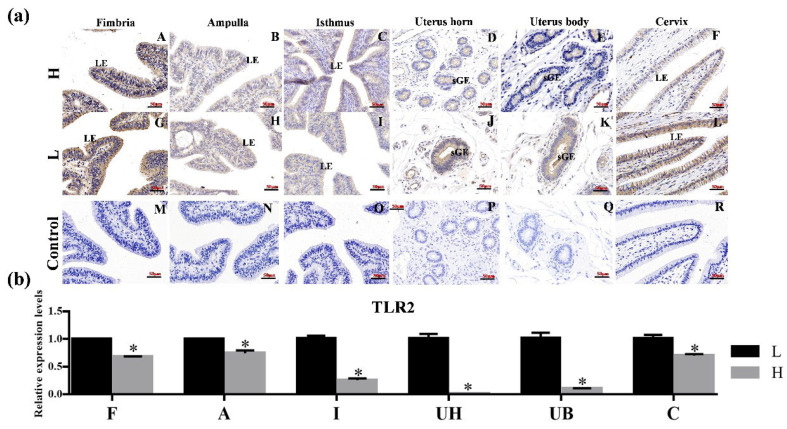
**The location and expression of TLR2 in the reproductive organs of high- and low-fecundity Hu sheep.** (**a**) Immunohistochemical comparison of TLR2 proteins in the fimbria, ampulla, isthmus, uterus horn, uterus body and cervix of the high- (H) and low (L)-fecundity sheep. (**A**–**F**): Immunohistochemical comparison of TLR2 proteins in the fimbria, ampulla, isthmus, uterus horn, uterus body and cervix of the high (H)-fecundity sheep; (**G**–**L**): Immunohistochemical comparison of TLR2 proteins in the fimbria, ampulla, isthmus, uterus horn, uterus body and cervix of the low (L)-fecundity sheep; (**M**–**R**): No specific staining was detected in the negative control sections. LE: luminal epithelium; sGE: superficial glandular; (**b**) The differential expression level of TLR2 in different organs of the high- (H) and low (L)-fecundity sheep. The experiment was biologically repeated three times (*n* = 3) and technically repeated three times for each group. The relative mRNA levels were normalized to GAPDH and the results were expressed relative to F group, with * representing significant differences (*p* < 0.05). F: fimbria tubae; A: ampulla of the oviduct; I: isthmus; UB: uterine body; UH: uterine horn; C: cervix.

As shown in Figure 6a, TLR2 was expressed in the endometrium of the uterine horns, uterine body and cervix. The staining intensity in the uterus bodies sGE of HP group was lower than that observed in the LP group (Figure 6a(E,K)). Additionally, TLR2 staining intensity in the LE of the HP cervix was also lower compared to its LP counterpart (Figure 6a(F,L)). However, no significant difference was observed in the three regions of the fallopian tube. No specific staining was detected in the negative control sections (Figure 6a(M–R)). The mRNA expression level of *TLR2* in the fimbria tubae, ampulla of the oviduct, isthmus, uterine horn, uterine body of the HP group was significantly lower than in the LP group (Figure 6b, *p* < 0.05).

**Figure 7:**
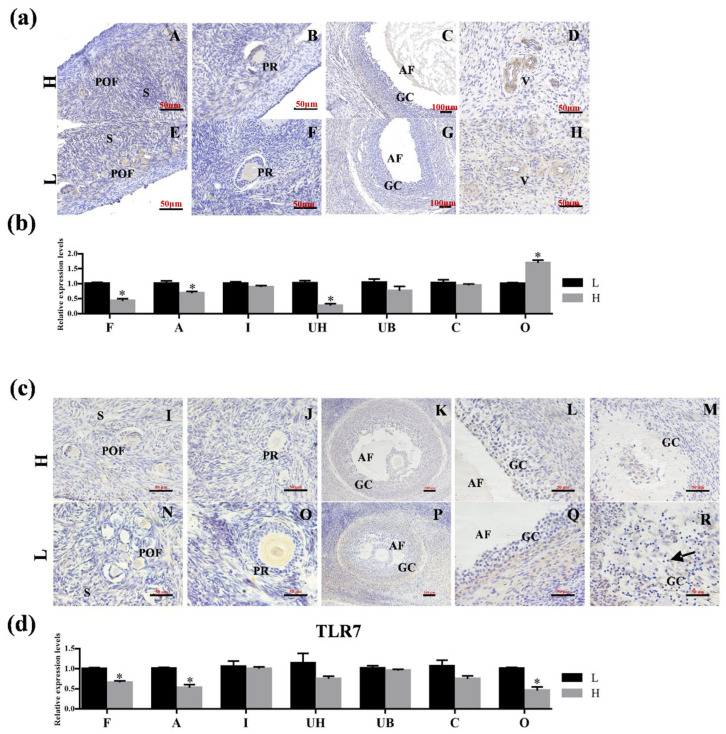
**The location and expression of TLR6 and TLR7 in the ovary of high- and low-fecundity Hu sheep.** (**a**,**c**) Immunohistochemical comparison of TLR6 and TLR7 proteins in the ovary of the high- (H) and low (L)-fecundity sheep. (**A**–**H**): Immunohistochemical comparison of TLR6 proteins in the ovary of the high- (H) and low (L)-fecundity sheep; (**I**–**R**): Immunohistochemical comparison of TLR7 proteins in the ovary of the high- (H) and low (L)-fecundity sheep. POF: primordial follicle; AF: antral follicle; GC: granulosa cell; PR: primary follicle; v: vessel. (**b**,**d**) The differential expression level of TLR6 and TLR7 in different organs of the high (H) and low (L) fecundity sheep. The experiment was biologically repeated three times (*n* = 3) and technically repeated three times for each group. The relative expression levels were normalized to GAPDH and the results were expressed relative to F group, with * representing significant differences (*p* < 0.05). F: fimbria tubae; A: ampulla of the oviduct; I: isthmus; UB: uterine body; UH: uterine horn; C: cervix; O: ovary.

The expression of TLR6 (Figure 7a) and TLR7 (Figure 7c) was evaluated in ovarian follicles at various stages in both the HP and LP groups. Notably, some antral follicles in the LP group exhibited nuclear pyrosis (Figure 7c(R)). As illustrated in Figure 7b, the expression of TLR6 was significantly higher (*p* < 0.05) in the LP ovaries, whereas TLR7 expression was significantly lower (*p* < 0.05) in the LP ovaries, as shown in Figure 7d.

## 4. Discussion

This study validates the hypothesis that Toll-like receptors (TLRs) exhibit specific expression patterns in the female reproductive system of Hu sheep, with certain TLRs potentially influencing fecundity. Our findings highlight the significant regulatory role of TLRs in reproductive organ function and their impact on the reproductive capacity of Hu sheep, an economically valuable breed known for its prolificacy.

Understanding the molecular mechanisms underlying reproduction is crucial for improving sheep-breeding outcomes. While previous research documented the expression of TLR1-9 in ewe tissues [16], our study provides a comprehensive characterization of their expression patterns across various reproductive organs in Hu sheep, with a particular focus on TLR2, TLR6, and TLR7 in relation to follicular development and fertility variations.

The ovary, uterus, and oviduct serve as primary reproductive organs and target sites for reproductive hormones. Evidence suggests significant interplay between the immune and reproductive systems involving TLRs [19,20,28]. Reproductive hormones likely influence reproductive and physiological functions by regulating TLR expression in these organs [29,30,31]. Notably, variations in luteinizing hormone levels have been observed among ewes with differing prolificacy [32], which prompted our investigation into TLR expression patterns across reproductive organs of Hu sheep with varying fertility levels.

Our results revealed organ-specific expression patterns of TLRs with potential functional implications. TLR2, TLR3, and TLR4 showed elevated expression in the oviduct and uterus, consistent with previous findings indicating their crucial roles as pattern recognition receptors in mouse oviducts [33,34] and in mediating virus-specific mucosal immunity in the fallopian tubes [35,36,37]. Additionally, TLR6 exhibited high expression in the oviduct, suggesting its significant contribution to oviduct function in Hu sheep. TLR7 demonstrated particularly high expression in the ovary, aligning with previous research indicating its involvement in the luteolytic mechanism of ovine corpus luteum [38]. Collectively, these results highlight TLR2, TLR6, and TLR7 as potential regulators of reproductive organ function influencing the reproductive capacity of Hu sheep.

The expression patterns of TLRs in the uterus closely resembled those in the oviduct. TLRs play indispensable roles in uterine pregnancy recognition and endometrial immune defense during the estrous cycle [39,40]. Notably, TLR3, TLR6, and TLR7 exhibited higher expression levels than other TLRs in the ovary, aligning with Liu et al.’s (2008) observation that ovulation involves the regulation of genes important in innate immune responses [19]. These findings collectively suggest that TLR2, TLR6, and TLR7 function as significant innate immune molecules in ovine reproduction.

Immunohistochemical analysis revealed distinct localization patterns and expression differences between high-prolificacy (HP) and low-prolificacy (LP) groups. TLR2 was primarily detected in stromal cells, luminal and glandular epithelia of the oviduct and uterus. In mammals, the oviduct facilitates oocyte transport and fertilization, with TLRs protecting cumulus–oocyte complexes (COCs) from pathogens in the reproductive tract [19,41,42] and protecting the uterus and oviduct from infectious agents [43,44]. The expression of TLR2 across various regions—including the ampulla, isthmus, fimbriae tubae, uterine horn, uterine body, and cervix—showed significantly lower levels in the HP group compared to the LP group. Previous research has documented TLR2 localization in the reproductive tract of various species, including turtles [45] and dogs, with heightened expression in ciliated cells, cilia, and secretory gland vesicles of the oviduct [46]. The significantly lower TLR2 expression in HP group oviducts, uteri, and ovaries suggests this receptor may regulate fertility-related processes associated with prolificacy.

Our analysis of TLR6 revealed its predominant localization in luminal epithelial (LE) and superficial glandular epithelial (sGE) cells of the uterus. This finding aligns with research showing that epithelial and stromal cells of bovine endometrium contribute to innate immunity by initiating inflammatory responses to bacterial lipopeptides via TLR6 [47], suggesting a similar role in Hu sheep uterine immunity. Additionally, TLR6 was detected in LE and circular muscle (CM) cells of the oviduct, potentially serving functions similar to those in the uterus, though this hypothesis requires further experimental validation. Interestingly, TLR6 expression was significantly higher in HP group ovaries compared to the LP group, suggesting that TLR6 may enhance reproductive outcomes by modulating ovarian function and providing immune protection to reproductive organs.

Regarding TLR7, our findings align with previous reports of its expression in human oviducts [48] and in uterine LE and sGE of ewes [49]. The comparable expression patterns between human and ovine oviducts suggest conserved functions across species [31]. In the reproductive tract, hormones modulate TLR function both in vivo and in vitro within the endometrium [50,51], with estrogens specifically enhancing TLR7-mediated functions in women [52]. This suggests that TLR7 may participate in regulating reproductive tract functions in ewes, potentially influencing reproductive capacity. Notably, TLR7 expression in the ovary significantly exceeded that in other reproductive organs, indicating a potentially critical regulatory role in ovarian function. Furthermore, TLR7 expression was significantly lower in HP group ovaries compared to LP group ovaries, suggesting that TLR7 may influence ovarian function under hormonal regulation.

Our examination of follicular development revealed no significant differences in TLR2, TLR6, and TLR7 expression across various developmental stages in Hu sheep ovaries. These receptors were detected in both theca and granulosa cells, suggesting their importance in follicle development. They may also be associated with extensive remodeling processes during ovulation, potentially directly affecting reproductive outcomes. Previous research has linked TLR2 with polycystic ovary syndrome and metabolic comorbidities in obese women [53], with TLR2 mRNA levels increasing from early to mid-luteal phase [54]. TLR6 and TLR7 have shown opposing expression trends during luteal dissolution induced by PGF2-α [54], mirroring our observed opposite expression patterns of these receptors in HP and LP group ovaries.

To our knowledge, this study represents the first systematic report of TLR2, TLR6, and TLR7 protein expression in follicles at different developmental stages in Hu sheep. While no significant difference in expression intensity was found between TLR6 and TLR7 in the ovaries, we observed TLR7 expression around apoptotic bodies of granulosa cells. This suggests that TLR7 may mediate granulosa cell apoptosis, thereby influencing follicular ovulation and ultimately affecting prolificacy.

## 5. Conclusions

The differential expression of TLR2, TLR6, and TLR7 between high- and low-prolificacy Hu sheep suggests a potential association between innate immunity and reproductive function, with TLR7’s localization around granulosa cell apoptotic bodies indicating a possible contributory role in follicular development. Our work provides an additional perspective on the molecular complexity of sheep reproduction, potentially informing future research that integrates immune-related mechanisms with the hormonal, nutritional, and genetic factors that collectively determine reproductive efficiency.

## Figures and Tables

**Table 1:** The primer sequences list of genes.

Gene	Primer Sequence 5′-3′	PCR Product Size (bp)	Annealing Temperature (°C)
*TLR1*	F-CCCAACTTTGTCCAGAGCGA	262	60
	R-CTGCTGCTTTTCCCATCAGTT		
*TLR2*	F-ACGGGCTGTGGTACATGAAG	212	60
	R-TTTGCCAGGGACAAGGTCTC		
*TLR3*	F-TTTTCTTGGTTGGGGCACCT	193	60
	R-CCACCCTTCGAAGCATCAGT		
*TLR4*	F-TCCACCTGATGCTTCTTGCT	203	60
	R-GATGATATTGGCGGCGATGG		
*TLR5*	F-GGGAGACTGCCTTGACCTTC	289	60
	R-GAGATTGGGCAGGTTTCGGA		
*TLR6*	F-TCCAATCACCACGAGTCTCA	158	60
	R-GCAAGTGAGCAACAGGTAGT		
*TLR7*	F-AAACTCTGCCCTGTGATGTC	148	60
	R-GAGATGCCTGCTATGTGGTT		
*TLR8*	F-CCCGAAGCTATCCTTGCGAT	294	60
	R-GCAGCAACTCCCTTAGGTGT		
*TLR9*	F-CTGCTGCTGTCCTACAACCA	244	60
	R-CGCGGAACCAGTCTTTCTCT		
*GAPDH*	F-GTCAAGGCAGAGAACGGGAA	232	60
	R-GGTTCACGCCCATCACAAAC		

*TLR1*: Toll-like receptor 1; *TLR2*: Toll-like receptor; *TLR3*: Toll-like receptor 3; *TLR4*: Toll-like receptor 4; *TLR5*: Toll-like receptor 5; *TLR6*: Toll-like receptor 6; *TLR7*: Toll-like receptor 7; *TLR8*: Toll-like receptor 8; *TLR9*: Toll-like receptor 9; *GAPDH*: glyceraldehyde 3-phosphate dehydrogenase. F: Forward primer R: Reverse primer.

## Data Availability

The original contributions presented in this study are included in this article. Further inquiries can be directed to the corresponding author.
